# The Newest “Omics”—Metagenomics and Metabolomics—Enter the Battle against the Neglected Tropical Diseases

**DOI:** 10.1371/journal.pntd.0003382

**Published:** 2015-02-12

**Authors:** Geoffrey A. Preidis, Peter J. Hotez

**Affiliations:** 1 Section of Gastroenterology, Hepatology & Nutrition, Department of Pediatrics, Baylor College of Medicine and Texas Children’s Hospital, Houston, Texas, United States of America; 2 National School of Tropical Medicine, Department of Pediatrics and Molecular Virology & Microbiology, Baylor College of Medicine, Houston, Texas, United States of America; 3 Sabin Vaccine Institute and Texas Children’s Hospital Center for Vaccine Development, Houston, Texas, United States of America; 4 James A. Baker III Institute for Public Policy, Rice University, Houston, Texas, United States of America; 5 Department of Biology, Baylor University, Waco, Texas, United States of America; University of Melbourne, AUSTRALIA

The metagenomics and metabolomics eras have dawned on the neglected tropical diseases, bringing new promise for the development of diagnostics, therapeutics, and vaccines.

## Introduction

The international Human Microbiome Project [1,2] trumpeted the coming of age of the field of metagenomics, the study of entire communities of microbes and their contributions to health and disease. Metagenomic analyses are most often undertaken by sequencing the bacterial 16S ribosomal RNA (rRNA) subunit or by whole metagenome shotgun sequencing, typically on a massively parallel pyrosequencing platform. These technologies have expanded the scope of traditional culture-dependent microbiological methods and have enhanced our understanding of the rich microbial communities that inhabit the intestine, skin, oral cavity, and genitourinary tract and how these commensal microbes interact with both pathogen and host.

In parallel, the field of metabolomics emerged as the systematic, nonbiased analysis of all low-molecular-weight small molecules, or metabolites, produced by a system in response to an environmental stimulus. Metabolites are secreted into body fluids by host and microbial cells, measured by mass spectrometry–based approaches, and aligned against libraries of known biochemicals. These techniques have been used to gain insights into mechanisms of pathogenesis and to identify new biomarkers of disease. Metabolomics also offers clues to the presence and function of microbes living deep within the small bowel that are difficult to sample directly and highlights the complex relationship between resident microbes, host metabolism, pharmacotherapeutic action, and relative health or disease.

Metagenomics and metabolomics are the two most rapidly advancing “omics” technologies and lie at either end of the “omics cascade” [3]; the former identifies the genetic potential of a community, whereas the latter reports the actual biology that produces a phenotype. These fields have enabled discoveries pertinent to a number of human conditions—namely, acute gastroenteritis, antibiotic-associated diarrhea, inflammatory bowel disease, irritable bowel syndrome, liver disease, undernutrition, and obesity—and have begun to shed new light on multiple aspects of the neglected tropical diseases. Moreover, there are exciting opportunities to now pair metagenomic and metabolomic data in order to gain new and unprecedented insights into the host–parasite relationship. Here, we explore the nascent metagenomic and metabolomic contributions to the diagnosis, pathogenesis, treatment, and prevention (including vector control) of neglected tropical diseases. We then look ahead to the full potential of the postgenomics era and consider how metagenomics and metabolomics could help in the control and elimination of these diseases.

### Metagenomics

Multiple methods are used to study complex intestinal microbial communities [4,5]. Until a few decades ago, culture-based techniques were the primary means of studying bacteria, and these methods remain central to understanding how bacteria interact with each other and their environment. However, the majority of intestinal microbes are not easily grown under standard culture conditions. Thus, culture-independent technologies enable exploration of the intestinal microbiome in its entirety. Today’s commonly employed methods fall under one of two categories, the first of which is sequencing the 16S ribosomal RNA gene subunit. The 16S subunit is unique to bacteria and contains both conserved regions that serve as universal polymerase chain reaction (PCR) primer binding sites and variable regions that allow for identification of operational taxonomic units, in many cases to the species level. The sequencing process typically involves isolation of nucleic acid from samples, amplification of bacterial DNA using universal 16S PCR primers, cloning, sequencing using Sanger, 454, or Illumina platforms, and alignment to libraries of known 16S sequences, such as the Ribosomal Database Project [6]. These methods are attractive because of their affordability and tendency to be highly automated. However, they detect only the bacterial members of the microbiome, often fail to achieve species-level taxonomic identification, and are subject to both PCR amplification bias (which might omit entire bacterial clades) and to copy-number variation of the 16S gene. Alternatively, whole metagenome or metatranscriptome shotgun (WMS) sequencing involves all nucleotides in a sample, identifying bacteria, along with any archae, viruses, and fungi that may be present, to a species or even strain level while also providing functional data based on genome content. Drawbacks of WMS include higher cost (in terms of both nucleotide number and resources needed to analyze the larger data set) and contamination with host nucleic acid. Both 16S and WMS methods have an inherent nucleic acid extraction bias because some bacteria are lysed far more easily than others. And although much faster than Sanger sequencing, the “next-generation” technologies align shorter reads and as a result are more prone to sequencing errors. Statistical analyses for both sequencing methods typically include tables of relative taxonomic abundance, alpha diversity or community richness, and beta diversity or similarity between samples, the latter of which is often depicted with principal coordinate plots.

Initial metagenomic studies revealed that the intestinal microbiome plays critical roles in the development of innate and adaptive immunity, nutrition and growth, immune tolerance, and susceptibility to infections [7,8]. More recent data suggest that in the short term diet and environmental interactions rapidly alter microbiome composition [9], but in the long term an individual’s microbiome remains remarkably stable over time [10], can differ dramatically among nationalities and cultures [11], and may be persistently shaped by early life events such as breast feeding [12] or enteric infections [13]. Bacterial pathogens such as *Salmonella*, *Shigella*, and *Escherichia coli* can drastically alter the composition of the intestinal microbiome [14] using an array of mechanisms that include secretion of bioactive antimicrobial molecules, modulation of the host immune system, colonization resistance, and niche exclusion [14–17]. Presumably through some or all of these mechanisms, acquisition of enteric pathogens early in life can increase the risk of morbidities that persist long after the pathogen has been eliminated from the body. Examples include severe acute malnutrition, lower IQ, diminished efficacy of live oral vaccines [18], postinfectious functional gastrointestinal disorders [19–21], and noncommunicable metabolic diseases such as obesity, cardiovascular disease, and type 2 diabetes [22].

**Fig 1:**
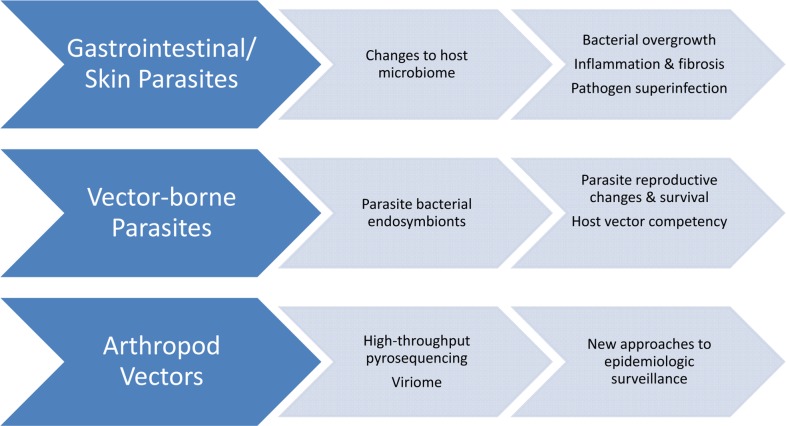
Neglected tropical diseases (NTDs) and the microbiome.

However, bacteria are not the only pathogens that reshape the microbiome (Fig. 1). In a model of *Trichuris suis* infection, whipworms do not cause overt diarrhea, but they do alter the relative abundance of 13% of genera in the porcine proximal colon. Even more dramatic changes were identified by WMS, which revealed that 26% of all metabolic pathways were altered by infection, most significantly replication, recombination and repair, carbohydrate transport and metabolism, and cell wall, membrane, and envelope biogenesis [23]. Likewise, *Opisthorchis viverrini* changes the fecal microbiome of hamsters at every taxonomic level from phylum to genus, including enrichment of Ruminococcaceae, Lachnospiraceae, and *Lactobacillus*. Stool from infected animals contained increased overall microbial loads and alpha diversity compared to controls. Unexpectedly, thousands of fluke-associated bacteria were detected in the bile of infected animals (bile was sterile in uninfected controls); among these biliary bacteria were 60 taxa not isolated in stool, including the potentially pathogenic genera *Bordetella*, *Burkholderia*, *Pseudomonas*, *Serratia*, and *Sphingomonas* [24]. These alterations potentially contribute to biliary inflammation and periductal fibrosis and ultimately to the sequence of events leading to neoplastic changes and cholangiocarcinoma. Finally, *Sarcoptes scabiei* modifies the porcine skin microbiome, increasing *Staphylococcus* colonization from <1% to up to 80% relative abundance following infestation with mites [25], potentially increasing the risk of bacterial superinfection. Nonetheless, not all helminths alter their microbial environment in all infections; no substantial changes to the fecal microbiome were detected in Ecuadorian school children infected with *Trichuris trichiura* [26] or in adults experimentally infected with *Necator americanus* [27]. Important next steps include identifying mechanisms by which alterations in the host microbiome mediate disease and proposing strategies by which the microbiome could be “repaired” to lessen the risk of long-term morbidity.

A parasite’s endosymbiotic bacteria can play integral roles in the pathogenesis of disease. This concept was demonstrated decades ago, with doxycycline eradication of *Wolbocchia* spp. in *Onchocerca volvulus* effectively sterilizing adult worms [28]. Metagenomic studies are now searching for similar relationships by exploring the microbiomes of insect vectors. In the mature tsetse fly gut, maternally transmitted endosymbiotic *Wigglesworthia* and *Sodalis* spp. are required to maintain viable African trypanosomes [29]. On the other hand, *Trypanosoma cruzi* reduces the total number of resident bacteria within the reduviid bug; this antibacterial effect, thought to be mediated by increased phenoloxidase activity that in turn enhances the reduviid bug’s innate immunity, is required for normal parasite development [30]. With the recent report of the multikingdom microbiome associated with the sandfly vector of *Leishmania infantum chagasi* [31], new strategies of pathogen elimination via vector control for both Chagas disease and visceral leishmaniasis may soon be proposed.

High-throughput pyrosequencing has been suggested as an improved means of detecting arthropod-borne viruses among entire populations of vectors, such as dengue virus detection in mosquitoes [32]. Sequencing the metagenome of multiple tick vectors revealed known tick-borne pathogens including *Anaplasma*, *Bartonella*, *Borrelia*, *Ehrlichia*, *Francisella*, and *Rickettsia*, as well as other potential pathogens, including members of the phylum Chlamydiae [33]. This technology could dramatically improve our current culture-based surveillance techniques. Similar methods were used to detect viral nucleic acid in sera from humans with dengue-like viral illnesses [34] and to identify the prevalence and spread of antibiotic resistance markers among bacteria in the human intestinal microbiome [35–38]. Finally, metagenomics has been used to gain insight into the spatiotemporal dynamics of particularly virulent strains of important bacterial pathogens, most notably for *Staphylococcus aureus* [39–42]. Analogous eukaryotic genomic approaches could be applied to monitor geographic spread and virulence in protozoans or to highlight selective pressures that drive human hookworm and schistosome populations to become resistant to antihelminthic drugs. Among the challenges to applying these techniques to eukaryotes is the relative difficulty of DNA isolation and the probable need to target a subset of the substantially larger eukaryotic genome to make population surveillance feasible on a grand scale. As improvements in sequencing and microbial identification develop, new methods of surveillance and diagnosis will merit testing in the field.

### Metabolomics

Metabolites produced by microbial and host cells contain an extraordinary array of physicochemical properties, may be present in virtually any body tissue or fluid, and are found in concentrations differing by multiple orders of magnitude. As a result, no single metabolomics platform is capable of detecting all metabolites in a sample, and a combination of approaches is typically employed [3,43]. Mass spectrometry coupled to gas chromatography (GC-MS) detects volatile, thermally stable metabolites with less than millimolar sensitivity, whereas liquid chromatography (LC-MS) is used to detect nonvolatile polar and nonpolar compounds with nanomolar resolution. Both of these approaches are dependent on sample preparation methods that introduce bias and inevitably metabolite losses. Alternatively, nuclear magnetic resonance (NMR) spectroscopy does not require prior separation of compounds within a sample, although minimizing sample preparation comes at a cost in terms of resolution; NMR can usually only detect compounds at or above a millimolar level. After alignment to libraries of known biochemicals, multivariate statistical approaches present metabolite contingency tables and principal component analyses. The major drawback to all metabolomics approaches is cost, both in terms of data acquisition and labor intensity of data analysis. Moreover, a fully annotated, comprehensive metabolite library, especially for microbial-derived compounds, is still many years away.

**Fig 2:**
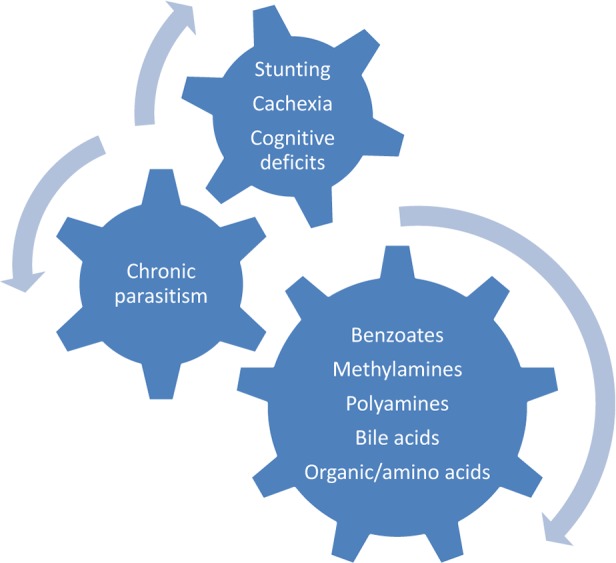
Metabolomic profiles in parasitism.

Early metabolomic studies revealed that small changes in the intestinal compartment have profound effects on host metabolites measured outside of the intestine. Manipulation of the mouse intestinal microbiome altered the metabolome in every tissue or biofluid analyzed [44,45]. Similarly, *Fasciola hepatica* infection in rats induced metabolic changes in areas as remote from the liver as brain tissue. Metabolic profile differences were manifest in altered concentrations of neural nucleotides; specifically, brains of infected rats had increased concentrations of inosine, tyrosine, and phenylalanine and decreased levels of glycerophosphocholine, succinate, inosine mono-, di-, and triphosphate, adenosine, and adenosine mono-, di-, and triphosphate compared to controls [46]. Such evidence could provide the basis for neurological sequelae of some helminth infections, even in the absence of direct invasion of brain parenchyma. For example, could metabolite alterations partially account for the cognitive disturbances linked to pediatric gastrointestinal nematode or schistosome infections in developing countries [47–49] or the common parasitic infections in North America linked to brain dysfunction [50]? Still another worthwhile avenue for metabolomic investigations is the potentially altered profiles in both humans and animals with wasting syndromes and growth deficits linked to some chronic helminth and protozoan infections, including hookworm and other gastrointestinal helminths, schistosomiasis, leishmaniasis, and trypanosomiasis (Fig. 2) [51–54].

In more readily accessible biofluids, metabolomics holds great potential to identify new biomarkers of disease. For example, a comprehensive urinary metabolic fingerprint distinguished mice infected with *Schistosoma mansoni* from controls; among the most significantly deranged metabolic pathways were tricarboxylic acid (TCA) cycle intermediates (suggestive of increased glycolysis), amino acid metabolism including depletion of taurine, and a number of microbial-derived biochemicals that suggest major perturbations to the microbiome [55]. Complete sets of urine and plasma metabolites from mice infected with *Schistosoma japonicum* were able to detect infection one week earlier than the current gold standard method, microscopic visualization of parasite eggs; the majority of significantly altered metabolites are included in the pathways outlined above [56]. Complete urinary metabolic profiles were also used to diagnose humans infected with *S*. *mansoni* [57], whereas complete urine, plasma, and stool profiles were used to identify mice infected with *Echinostoma caproni* [58]. It should be noted that most metabolomic animal studies have been performed with inbred mice, so the generalizability of individual biomarkers to either outbred mice or humans is unknown. In one illustrative example, metabolomic analysis of BALB/c mice infected with *S*. *aureus* revealed 167 blood metabolites significantly discriminating animals given adequate therapy from those given inadequate antibiotic therapy. Only 25 of these metabolites were among the 126 significantly altered metabolites comparing acute versus antibiotic-treated intensive care unit patients with *S*. *aureus* sepsis. Nonetheless, a subset of the 25 metabolites common to inbred mouse and clinical studies successfully predicted treatment response in humans [59].

Ideally, diagnoses would be made using more selective subsets of metabolites. In humans, a set of 14 serum- and plasma-derived biomarkers accurately identified humans infected with *O*. *volvulus* (four proteins, two fatty acids/sterol lipids, two sterol lipids, and one hexacosenoic acid, pentacosenoic acid, fatty alcohol/adehyde, fatty acid, hydroxy-octadecenoic acid, and phosphorylated sphingolipid) and further distinguished individuals with viable worms from those harboring worms of decreased virulence after ivermectin therapy [60]. Finally, metabolomics revealed a single biomarker in human urine that is specific to infection with *O*. *volvulus*: biochemical studies revealed that *N*-acetyltyramine-*O*,β-glucuronide (NATOG) is derived from the neurotransmitter tyramine, metabolized by the nematode, and is a marker of parasite viability [61]. There is an urgent need for new and improved diagnostics for most of the parasitic infections of humans. Identification of a few key biomarkers could ultimately replace more expensive approaches to diagnosis and disease monitoring while lending new insights into the pathogenesis of other neglected tropical diseases.

Metabolomic techniques are being employed not only to diagnose disease but also to monitor disease course and determine prognosis. For human dengue virus, serum metabolic signatures specific to various stages of infection have been identified. Metabolites significantly elevated in infected patients include free fatty acids, acylcarnitines, phospholipids, and amino acids that participate in a surprisingly diverse range of pathways such as fatty acid biosynthesis and beta-oxidation, phospholipid catabolism, and steroid hormone metabolism [62]. Among the lipid metabolites are biomarkers that show promise as early prognostic indicators [62]. Similarly, three polyunsaturated fatty acid biomarkers, among them arachidonic acid, distinguish patients with low bacterial indices of *Mycobacterium leprae* from those with high bacterial indices [63] in leprosy, a disease in which lipid turnover is believed to mediate pathogenesis in high-burden lesions [64]. Finally, autopsy specimens revealed abnormal patterns of inflammation and anaerobic metabolism in the brain of an elderly individual who died despite receiving the standard postexposure neuroprotection for rabies, the strain of which was identified using deep sequencing of host RNA [65]. An important caveat regarding comparative metabolomic studies is that biomarkers are typically identified by comparing infected versus uninfected hosts, rather than hosts infected by one versus another pathogen. Therefore, it is unclear to what extent the reported metabolic derangements represent a pathogen-specific host response or a more general response to a morbid state.

Perhaps the greatest immediate impact of metabolomics in the battle against the neglected tropical diseases will be borne through in vitro studies that enhance our understanding of basic mechanisms of parasite metabolism and drug resistance. These approaches have already been used to reveal mechanisms of action of antiparasitic drugs, such as benznidazole binding to thiol groups to mediate toxicity against *T*. *cruzi* [66], and mechanisms of antimicrobial resistance, such as alteration of *L*. *donovani* membrane proteins as a means of resistance to antimonial drugs [67]. Metabolomic techniques revealed a novel antioxidative glycerol biosynthetic mechanism that helps *Entamoeba histolytica* evade host defenses during infection [68], and other studies revealed a lack of synergy between two commonly used trypanocidal drugs [69]. Currently, there is particular interest in the identification of novel aspects of carbon utilization by parasites. For example, a canonical, oxidative TCA cycle that depends upon the mitochondrial branched chain ketoacid dehydrogenase complex [70] allows *Toxoplasma gondii* to survive within host cell vacuoles and is essential for parasite reproduction [71]. Similarly, *L*. *mexicana* uses a novel TCA cycle that depends on mitochondrial aconitase and glutamine synthetase; this energy cycle is required for amastigotes to differentiate to their intracellular form [72]. As numerous recent discoveries pertaining to carbon metabolism in *Plasmodium* [73–75] have injected new energy into the drug development pipeline for malaria, each newly identified parasitic pathway of carbon utilization presents a novel potential therapeutic target for a neglected tropical disease.

### Future Perspectives

By any measure, the fields of metagenomics and metabolomics remain in their infancy. Current methods of analyzing multidimensional data sets lag behind advances in detection techniques. Much of the genetic material extracted from samples cannot yet be attributed to known microbes or to specific functions, and many of the metabolites are of unknown origin and function. Nonetheless, as comprehensive reference sets of nucleic acid sequences and biochemicals are expanded, the yield from these newest “omics” studies will improve.

Future studies will seek to understand how the intestinal microbiome functions as a metabolic organ and how to modify portions of the microbiome in order to strengthen host immunity. The influence of nonbacterial microorganisms, including the mycobiome and viriome (eukaryotic as well as prokaryotic viruses), should not be underestimated [76]. In one example, a phage-encoded virulence factor, sasX, helped enable a recent methicillin-resistant *S*. *aureus* epidemic in Asia by enhancing nasal colonization, immune evasion, and pulmonary abscess formation [77]. Additional studies using metabolomics might identify low-cost biomarkers from easily accessible body fluids that indicate infection, therapeutic efficacy, or drug resistance. Alternately, small-molecule therapeutics may target specific metabolic pathway deficiencies that contribute to certain disease states. Successful endeavors might need to employ multiple steps along the “omics” cascade (metagenomics, transcriptomics, proteomics, and metabolomics) simultaneously to increase the yield of exploratory studies. One currently available resource that helps catalog these enormous data sets is the freely accessible LeishCyc database. Launched as a compilation of *L*. *major* genes, LeishCyc has evolved to house a comprehensive bank of gene products, metabolites, and biochemical pathways from transcript, protein, and metabolome profiling studies in an integrated format [78].

Finally, we should seek to understand the enormous contribution of nutrition to the pathogenesis of the neglected tropical diseases through the lenses of metagenomics and metabolomics. Undernutrition has recently taken center stage in microbiome research, with recent studies revealing a potentially pathologic intestinal microbial profile associated with severe acute malnutrition [79] along with the ability of antibiotics to help restore nutritional status in the undernourished host [80]. Other studies have begun to define the metabolome of the undernourished host in laboratory models [81,82], and we are now beginning to link these profiles back to the metagenomic data. We ultimately seek to understand how specific microbes contribute to nutritional status, how metabolic profiles contribute to disease pathogenesis, and how manipulation of either may influence the host response to infection.

Metagenomics and metabolomics have already begun to enhance our understanding of the neglected tropical diseases. As both sciences continue to evolve, their direct clinical impact in the battle against these pathogens draws near.
